# Integration of host gene regulation and oral microbiome reveals the influences of smoking during the development of oral squamous cell carcinoma

**DOI:** 10.3389/fonc.2024.1409623

**Published:** 2024-10-15

**Authors:** Dan Liang, Xuemeng Ma, Xiaoyi Zhong, Yinghua Zhou, Wenxia Chen, Xuan He

**Affiliations:** ^1^ Guangxi Clinical Research Center for Craniofacial Deformity, Guangxi Medical University, Nanning, China; ^2^ Guangxi Key Laboratory of Oral and Maxillofacial Rehabilitation and Reconstruction, Guangxi Medical University, Nanning, China; ^3^ The Department of Operative Dentistry and Endodontology, College of Stomatology, Hospital of Stomatology, Guangxi Medical University, Nanning, China; ^4^ Department of Oral Pathology, College of Stomatology, Hospital of Stomatology, Guangxi Medical University, Nanning, China; ^5^ Comprehensive Care Clinic, College of Stomatology, Guangxi Medical University, Nanning, China

**Keywords:** oral squamous cell carcinoma, cigarette smoking, gene regulation, oral microbiome, host-microbe interactions

## Abstract

**Objective:**

This study aims to investigate the regulation of host gene transcription and microbial changes during the development of oral squamous cell carcinoma (OSCC) associated with smoking.

**Methods:**

The OSCC mouse model and smoking mouse model were established using 200 μg/mL 4-nitroquinoline-1-oxide (4NQO) in drinking water and exposure to cigarette smoke (four cigarettes per session, once a day, 5 days a week). Tongue tissues were harvested at 4 weeks and 16 weeks. Histopathological changes were evaluated using hematoxylin and eosin staining and Ki67 staining. RNA sequencing was performed on the mouse tongue tissues to identify differentially expressed genes (DEGs), and the results were validated by RT-PCR and immunohistochemistry. 16S rDNA sequencing was used to analyze changes in the oral microbiota during the early development of OSCC, identifying differentially abundant taxa associated with smoking. Finally, associations between the relative abundances of the oral microbiome and host gene expression were modeled using the Origin software.

**Results:**

DEGs associated with smoking during the development of OSCC were identified. There were 12 upregulated genes, including NR4A3 and PPP1R3C, and 23 downregulated genes, including CD74 and ANKRD1. These genes were enriched in functions related to the signal transduction of cellular processes such as inflammation, differentiation, immunity, and PI3K/AKT, NF-κB signaling pathways. 4NQO and smoking treatment decreased oral microbial diversity and reduced the abundance of Bacteroidetes, Proteobacteria, and *Lactobacillus* but increased the abundance of *Staphylococcus*. Integrative analysis showed that the expression of CD74 was positively correlated with the relative abundance of *Lactobacillus*, while PPP1R3C was negatively correlated with Bacteroidota.

**Conclusion:**

In addition to characterizing host gene expression and the oral microbiome, our study explored the potential role of host–microbiome interactions in the development of OSCC. These findings enhance our understanding of smoking-related OSCC occurrence and development, providing new insights for its prevention.

## 1 Introduction

Annually, approximately 300,000 new cases of oral cancer are diagnosed worldwide, with a 5-year survival rate of 50%–60% (1, 2). Oral squamous cell carcinoma (OSCC) is an invasive epithelial tumor, accounting for 90% of oral cancer cases, and is characterized by varying degrees of differentiation. It leads to disfigurement and functional impairments, including difficulties with speech, taste, and swallowing, which severely impact the quality of life of patients (3). The development of OSCC is a dynamic process influenced by both genetic and environmental factors. Smoking is a key risk factor for OSCC (4), with smokers having a three to five times higher risk of developing the disease compared to non-smokers (5). The mechanism by which smoking promotes OSCC involves hypoxia, inflammation, and immune regulation (6, 7). Additionally, cigarette smoke condensates have been shown to increase the invasion and metastasis of OSCC (8). These findings underscore the significant role of smoking in the occurrence and progression of OSCC.

Research has shown that 16% of cancer cases are linked to microbial infections (9). A large number of microorganisms inhabit the human oral cavity. The hosts and oral microbiome maintain a dynamic balance through various bidirectional communication and regulation mechanisms. When microecological imbalances occur, changes in immune and metabolic signaling can influence cancer characteristics, potentially leading to oral cancer (10). High-throughput sequencing has demonstrated a strong correlation between OSCC and dysregulated oral bacteria (11, 12). Studies have begun to investigate changes in the oral microbiome during the development of OSCC, reporting many potential carcinogenic mechanisms, including excessive inflammatory response, promotion of malignant transformation, host immunosuppression, anti-apoptotic activity, and secretion of carcinogens such as acetaldehyde (13, 14).

Studies have demonstrated that smoking alters the composition of the oral microbiome, which is a primary environmental factor influencing oral pathophysiology. Toxic components and bacteria in cigarettes directly or indirectly affect the oral microbiome through hypoxia, immunosuppression, and biofilm formation, resulting in the loss of beneficial species and the colonization of pathogens, eventually leading to diseases (15, 16). However, the relationship between host gene transcription and oral microbial regulation in the context of OSCC development due to smoking is rarely reported. In a previous study, our group analyzed the microbial composition of OSCC tissues and distant normal tissues. We found that the bacterial diversity and abundance in OSCC tissues were higher than in normal tissues. Moreover, the prevalence of *Fusobacterium* in OSCC was significantly higher than in distant normal tissues, and its high expression was associated with smoking (17).

This study aimed to investigate the regulation of host gene transcription and microbial changes in smoking-related OSCC. RNA sequencing (RNA-Seq) and 16S rDNA sequencing were employed to characterize host gene expression and the oral microbiome. Subsequently, the potential role of host–microbiome interactions in the development of smoking-associated OSCC was explored.

## 2 Materials and methods

### 2.1 Establishment of smoking model and OSCC model

**Figure 1:**
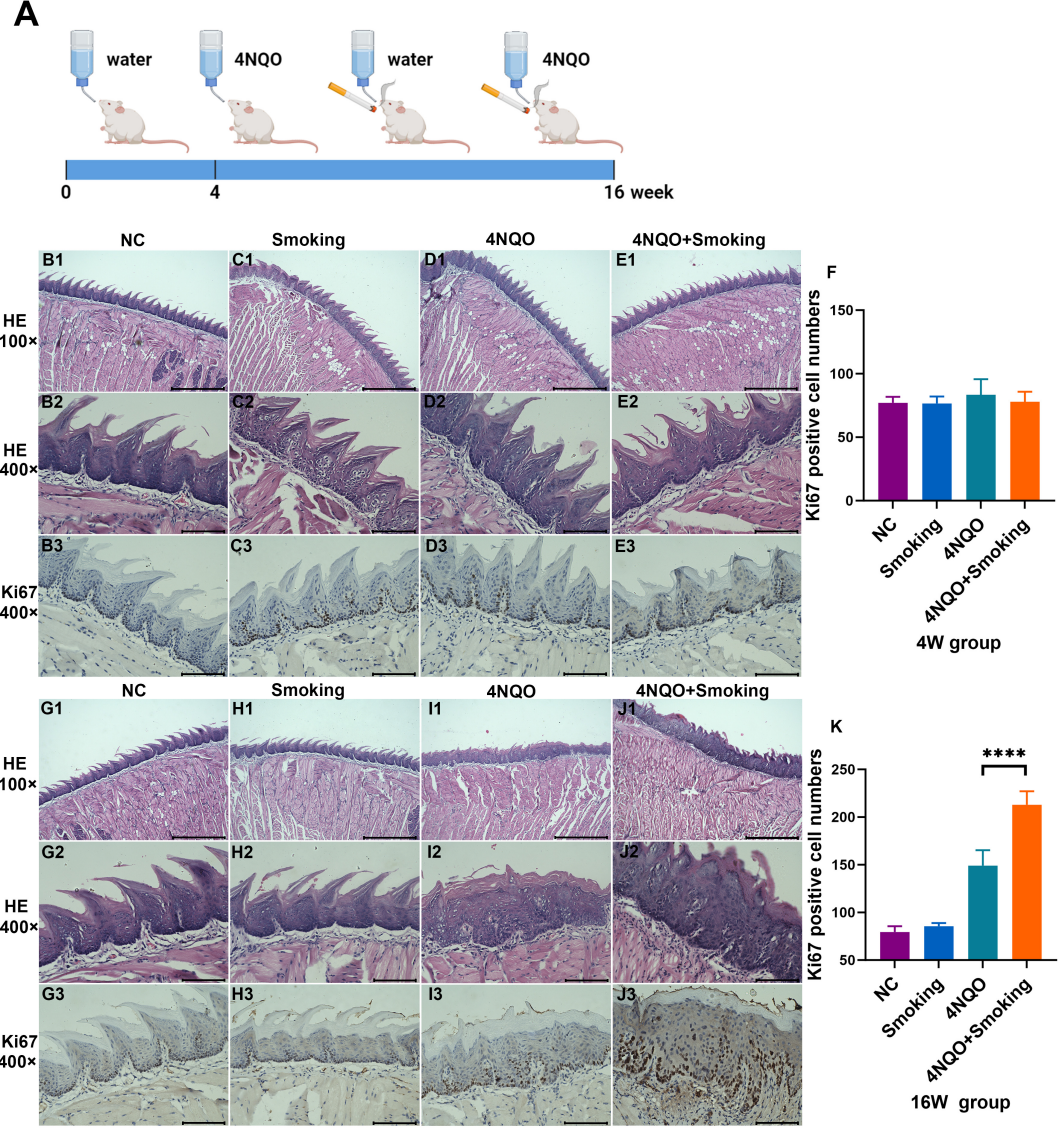
**(A)** Timeline for 4NQO and Smoking induction of OSCC. 5-week-old mice were treated with 4NQO and/or exposed to smoking for 4 or 16 weeks. Mice were euthanized at 4 weeks or 16 weeks to harvest tissue for analysis. Figure 1A was created with Biorender.com and has been granted permission. **(B–E)** H&E staining and Ki67 staining in NC group **(B)**, Smoking group **(C)**, 4NQO group **(D)**, and 4NQO+Smoking group **(E)** in 4 weeks. **(F)** Bar chart quantifying the Ki67-positive cell numbers in the four groups at 4 weeks. **(G–J)** H&E staining and Ki67 staining in NC group **(G)**, Smoking group **(H)**, 4NQO group **(I)**, and 4NQO+Smoking group **(J)** in 16 weeks. **(K)** Bar chart quantifying the Ki67-positive cell numbers in the four groups at 16 weeks. **(B1–E1, G1–J1)** Scale bar = 500 μm. **(B2–E3, G2–J3)** Scale bar = 100μm. ****p<0.0001 vs. control. 4NQO, 4-nitroquinoline-1-oxide; OSCC, oral squamous cell carcinoma.

All animal studies were approved by the Animal Ethics and Welfare Committee of Guangxi Medical University (202210909). A total of 80 five-week-old male BALB/C mice were purchased from Guangdong Weitong Lihua Co., Ltd. [SCXK(Guangdong)2022-0063]. The mice were randomly assigned to four groups (N = 10): a) negative control (NC) group, (b) Smoking group, c) 4-nitroquinoline-1-oxide (4NQO) group, and d) 4NQO+Smoking group (**Figure 1A**). Mice in the 4NQO and 4NQO+Smoking groups were given 4NQO water at a concentration of 200 μg/mL, with the water being changed weekly. Due to the lack of a peristaltic pump device, the mice were placed in a specialized smoking device (Chinese utility model patent number: ZL202223479539.9) (**Supplementary Figure 1**). The mice in the 4NQO+Smoking group and the Smoking group were exposed to cigarette smoke once daily (starting at 8 a.m.), with four cigarettes each time. The cigarettes were purchased from Nanning Cigarette Factory, with a tar content of 10 mg/20 cigarettes. Initially, two cigarettes were lit, and after 1 hour of exposure, there was a 10-minute ventilation break before lighting the remaining two cigarettes (7, 18). This exposure regimen was conducted for 5 days a week over a period of 4 or 16 weeks. The tongue tissues were harvested at 4 and 16 weeks, with the tongues bisected along the longitudinal axis. One half was fixed in 10% formalin, and the other half was frozen at −80°C.

### 2.2 Histological analysis

After fixation with formalin, tongue tissues were embedded in paraffin wax, sectioned, and stained with hematoxylin and eosin (H&E). The cell composition and distribution were observed under a light microscope. To investigate tumor cell proliferation, immunohistochemistry (IHC) staining was performed using a mouse anti-Ki67 monoclonal antibody. Ki67 positivity was indicated by yellow or brownish-yellow staining in the nucleus, and the number of positive cells was counted in a ×400 field of view. To identify protein molecules potentially associated with smoking-related OSCC, IHC staining was conducted using mouse anti-NR4A3 monoclonal antibody and anti-CD74 monoclonal antibody.

### 2.3 RNA-Seq

#### 2.3.1 RNA extraction and library construction

The experimental procedure was completed by Hangzhou Lianchuan Biotechnology Co., Ltd. Total RNA of tongue tissue was extracted using TRIzol reagent (Invitrogen, Carlsbad, CA, USA) following the manufacturer’s instructions. The quantity and purity of RNA from each sample were measured using a NanoDrop ND-1000 spectrophotometer (NanoDrop, Wilmington, DE, USA). RNA integrity was assessed using the Bioanalyzer 2100 (Agilent, Santa Clara, CA, USA) and confirmed to have an RNA integrity number (RIN) >7.0 by electrophoresis on a denaturing agarose gel. Poly(A) RNA was purified from 1 μg of total RNA using Dynabeads Oligo (dT)25-61005 (Thermo Fisher, Vacaville, CA, USA) through two rounds of purification. Subsequently, the poly(A) RNA was fragmented into small pieces using the Magnesium RNA Fragmentation Module (cat. e6150, NEB, Ipswich, MA, USA) at 94°C for 5–7 min. The fragmented RNA was then reverse-transcribed into cDNA using SuperScript™ II Reverse Transcriptase (Invitrogen, cat. 1896649, USA). Second-strand cDNA was synthesized using E. coli DNA polymerase I (cat. m0209, NEB, USA), RNase H (cat. m0297, NEB, USA), and dUTP Solution (cat. R0133, Thermo Fisher, USA). An A-base was added to the blunt ends of each strand to prepare them for adapter ligation. Indexed adapters with T-base overhangs were ligated to the A-tailed fragmented DNA. Single- or dual-index adapters were ligated to the fragments, followed by size selection using AMPure XP beads. The U-labeled second-stranded DNAs were treated with the heat-labile UDG enzyme (cat. m0280, NEB, USA). The ligated products were then amplified by PCR under the following conditions: initial denaturation at 95°C for 3 minutes; 8 cycles of denaturation at 98°C for 15 seconds, annealing at 60°C for 15 seconds, and extension at 72°C for 30 seconds; and a final extension at 72°C for 5 minutes. The average insert size for the final cDNA library was 300 ± 50 bp. Finally, 2 × 150 bp paired-end sequencing (PE150) was performed on an Illumina NovaSeq™ 6000 (LC-Bio Technology Co., Ltd., Hangzhou, China) according to the manufacturer’s protocol.

#### 2.3.2 Bioinformatics analysis

The Fastp software (https://github.com/OpenGene/fastp) was utilized to remove reads containing adaptor contamination, low-quality bases, and undermined bases using default parameters. The sequence quality was subsequently verified using Fastp. HISAT2 (https://ccb.jhu.edu/software/hisat2) was employed to map the reads to the Mus musculus reference genome, Ensembl_v101. The mapped reads for each sample were assembled using StringTie (https://ccb.jhu.edu/software/stringtie) with default parameters. All transcriptomes from all samples were then merged to reconstruct a comprehensive transcriptome using gffcompare (https://github.com/gpertea/gffcompare/). After generating the final transcriptome, StringTie was used to estimate the expression levels of all transcripts. Specifically, StringTie calculated expression levels for mRNAs using FPKM (FPKM = [total_exon_fragments/mapped_reads (millions) × exon length (kb)]). Differentially expressed mRNAs were identified with a fold change >2 or <0.5 and a parametric F-test comparing nested linear models (p-value <0.05) using the R package edgeR (https://bioconductor.org/packages/release/bioc/html/edgeR.html).

### 2.4 RT-PCR

Total RNA was extracted from tongue tissues using TRIzol reagent, and the purity and concentration of RNA were assessed. PrimeScrip™ RT reagent (Takara, Tokyo, Japan) was used to reverse transcribe the RNA into cDNA. The 2×SYBR Green qPCR Master Mix kit ∏ (Seven, Beijing, China) was employed for real-time fluorescence quantitative PCR amplification using the QuantStudio 5 system (Thermo Fisher Scientific, Waltham, MA, USA). The RT-PCR mixture consisted of 2×SYBR Green qPCR MasterMix (10 μL), primer forward (1 μL), primer reverse (1 μL), H_2_O (7 μL), and 1 μL transcribed cDNA. The protocol was as follows: initial denaturation at 95°C for 2 minutes; 40 cycles of 95°C for 15 seconds and 60°C for 1 minute; followed by a melting curve analysis with heating at 1.6°C/s to 95°C, 95°C for 15 seconds, 60°C for 1 minute, and 95°C for 1 second. Gene expression was analyzed using the 2^−ΔΔCT^ method. Primer sequences are listed in **Table 1**.

**Table 1:** Sequences for PCR amplification.

Gene	Primer sequences (5′–3′)
NR4A3	F: CGCCGAAACCGATGTCAGTA
	R: CTGCGAGGGCTCCTGTTGTA
CD74	F: CTTGCTGATGCGTCCAATGTC
	R: TCCTGGGTCATGTTGCCGTA
PPP1R3C	F: TTTGCAAGATCGGACGGTGA
	R: CAAAGGTGATCCGGACCTGAA
ANKRD1	F: TTTCTGAAAGCTGCGCTGGA
	R: TCTAAGCATGCTCGGTGGAGTG
β-Actin	F: CATCCGTAAAGACCTCTATGCCAAC
	R: ATGGACCACCATCCACA

### 2.5 16S rDNA sequencing

#### 2.5.1 DNA extraction and library construction

The experimental procedure was completed by Hangzhou Lianchuan Biotechnology Co., Ltd. Total DNA was extracted using the cetyltrimethylammonium bromide (CTAB) method and purified with AMPure XP beads (Beckman Coulter Genomics, Danvers, MA, USA). The DNA was amplified by PCR with primers targeting the V3–V4 region of the bacterial 16S rDNA gene (forward primer 341F: 5′-CCTACGGGNGGCWGCAG-3′, reverse primer 805R: 5′-GACTACHVGGGTATCTAATCC-3′). The 5′ ends of the primers were tagged with specific barcodes for each sample and sequencing universal primers. PCR amplification was conducted in a total volume of 25 μL, containing 25 ng of template DNA, 12.5 μL of PCR Premix, 2.5 μL of each primer, and PCR-grade water to adjust the volume. The PCR conditions for amplifying the prokaryotic 16S fragments included an initial denaturation at 98°C for 30 seconds; 32 cycles of denaturation at 98°C for 10 seconds, annealing at 54°C for 30 seconds, and extension at 72°C for 45 seconds; followed by a final extension at 72°C for 10 minutes. The PCR products were confirmed via 2% agarose gel electrophoresis. Throughout the DNA extraction process, ultrapure water, instead of a sample solution, was used as a negative control to exclude the possibility of false-positive PCR results. The PCR products were purified using AMPure XP beads (Beckman Coulter Genomics, Danvers, MA, USA) and quantified using Qubit (Invitrogen, USA). The amplicon pools were prepared for sequencing, and the size and quantity of the amplicon library were assessed using the Agilent 2100 Bioanalyzer (Agilent, USA) and the Library Quantification Kit for Illumina (Kapa Biosciences, Woburn, MA, USA), respectively. The libraries were sequenced on the NovaSeq PE250 platform according to the manufacturer’s recommendations and provided by LC-Bio Technology Co., Ltd.

#### 2.5.2 Bioinformatics analysis

Paired-end reads were assigned to samples based on their unique barcode and truncated by cutting off the barcode and primer sequences. Paired-end reads were merged using Fast Length Adjustment of SHort reads (FLASH). Quality filtering on the raw reads was performed under specific conditions to obtain high-quality clean tags according to fqtrim (v0.94). Chimeric sequences were filtered using the Vsearch software (v2.3.4). After dereplication using DADA2, a feature table and feature sequences were obtained. Alpha diversity and beta diversity were calculated by normalizing to the same sequences randomly. According to the SILVA (release 138) classifier, feature abundance was normalized using the relative abundance of each sample. Alpha diversity, analyzing species diversity complexity for a sample, was assessed using five indices: Chao1, observed species, Good’s coverage, Shannon, and Simpson. All these indices were calculated using QIIME2. Beta diversity was calculated using QIIME2, and the graphs were drawn using the R package. Blast was used for sequence alignment, and feature sequences were annotated using the SILVA database for each representative sequence. Other diagrams were implemented using the R package (v3.5.2). Differences in the relative abundance of species between the two groups were analyzed using the Wilcoxon rank-sum test and were considered significant at p < 0.05.

### 2.6 Integrated analysis of interactions between host gene and the oral microbiome

A correlation analysis was conducted between host gene expression data and oral microbiome abundance data at both the phylum and genus levels. The allometric function in Origin 2021 was employed to analyze differentially expressed genes (DEGs) and differential bacteria. Non-linear curve fitting was performed to examine the correlations between these variables. The R^2^ value was used to assess the degree of correlation, where a high R^2^ value indicates a stronger correlation between the two datasets.

### 2.7 Data analysis

Data analysis was conducted using GraphPad Prism 10.0. Data following a normal distribution are expressed as mean values ± standard deviations (SD), and comparisons between two groups were performed using Student’s t-test. Data not following a normal distribution are presented as median values, and the Wilcoxon rank-sum test was used for statistical analysis. A p-value <0.05 was considered statistically significant.

## 3 Results

### 3.1 Smoking promotes the development of OSCC

The mice were treated with smoking and/or 4NQO water. At 4 weeks, no morphological or histological changes were observed in the mucosa (**Figures B1–3, C1–3, D1–3, E1–3**). Ki67 staining showed no significant difference in the number of positive cells in tongue tissue among the four groups (**Figure 1F**). At 16 weeks, pathological examination revealed mild-to-moderate epithelial dysplasia appearing in the 4NQO and 4NQO+Smoking groups, with a few cases of severe dysplasia. Epithelial keratosis increased and epithelial pegs decreased in the 4NQO group and 4NQO+Smoking group (**Figures G1–3, H1–3, I1–3,J1–3**). The number of Ki67-positive cells in the 4NQO+Smoking group was significantly higher than in the group treated with only 4NQO, indicating a higher proportion and more severe epithelial dysplasia in the 4NQO+Smoking group (**Figure 1K**).

### 3.2 Transcriptome profiling to investigate gene expression changes

#### 3.2.1 Data quality control

The average number of raw reads obtained from all samples was M ± N. After adapter trimming, quality control, and chimera filtering, the average number of valid reads was m ± N, with an average validity rate of M. The percentages of Q20% and Q30% were above 99% and 97%, respectively. The average mapping rate to the reference genome exceeded 74%.

#### 3.2.2 Analysis of gene expression differences at 4 weeks

**Figure 2:**
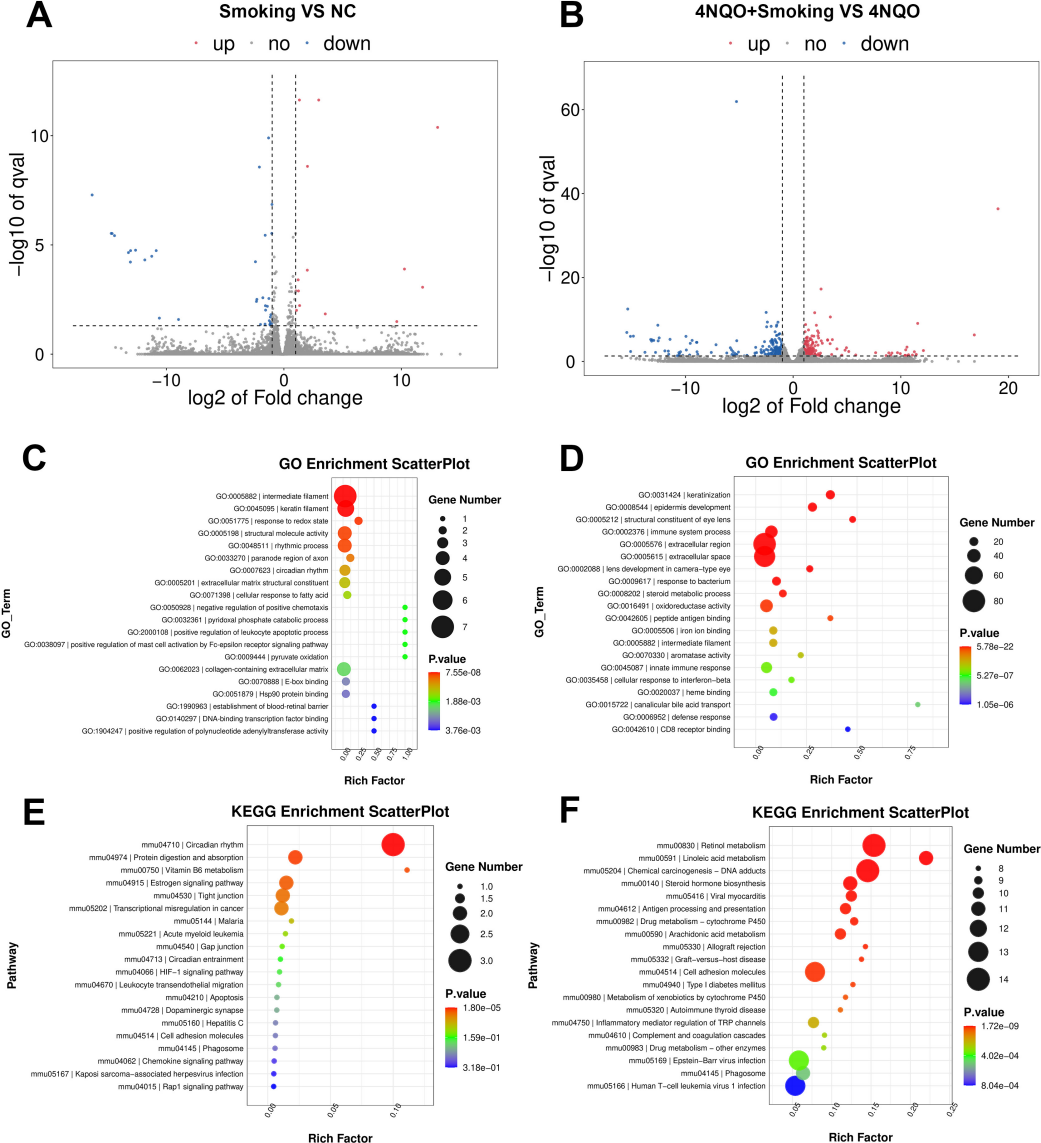
Four-week RNA sequencing identified differentially expressed mRNAs between the groups. **(A, B)** Transcriptome volcano map. Differentially expressed genes between groups were identified using a default threshold of p < 0.05 and |log2FC| ≥ 1. The identified genes were then visualized on a map to represent their expression levels. Blue indicates downregulated genes, and red indicates upregulated genes. **(A)** Smoking vs. NC group and **(B)** 4NQO+Smoking vs. 4NQO group. **(C)** GO functional enrichment analysis in Smoking vs. NC group. **(D)** GO functional enrichment analysis in 4NQO+Smoking vs. 4NQO group. **(E)** KEGG enrichment analysis in Smoking vs. NC group. **(F)** KEGG enrichment analysis in 4NQO+Smoking vs. 4NQO group. 4NQO, 4-nitroquinoline-1-oxide; GO, Gene Ontology; KEGG, Kyoto Encyclopedia of Genes and Genomes.

In the 4-week groups, 14 upregulated genes and 35 downregulated genes were identified in the Smoking vs. NC group. In the 4NQO+Smoking vs. 4NQO group, 236 upregulated and 232 downregulated genes were identified (**Figures 2A, B**). Gene Ontology (GO) enrichment analysis revealed that DEGs in the Smoking vs. NC group were primarily enriched in intermediate filament, keratin filament, and response to redox state (**Figure 2C**). In the 4NQO+Smoking vs. 4NQO group, DEGs were mainly enriched in keratinization, epidermis development, and immune system process (**Figure 2D**). Kyoto Encyclopedia of Genes and Genomes (KEGG) pathway analysis showed significant enrichment in Circadian rhythm, Protein digestion and absorption, and Vitamin B6 metabolism pathways in the Smoking vs. NC group (**Figure 2E**). In the 4NQO+Smoking vs. 4NQO group, DEGs were mainly involved in Retinol metabolism, Linoleic acid metabolism, and Chemical carcinogenesis DNA adducts (**Figure 2F**).

#### 3.2.3 Analysis of gene expression differences at 16 weeks

**Figure 3:**
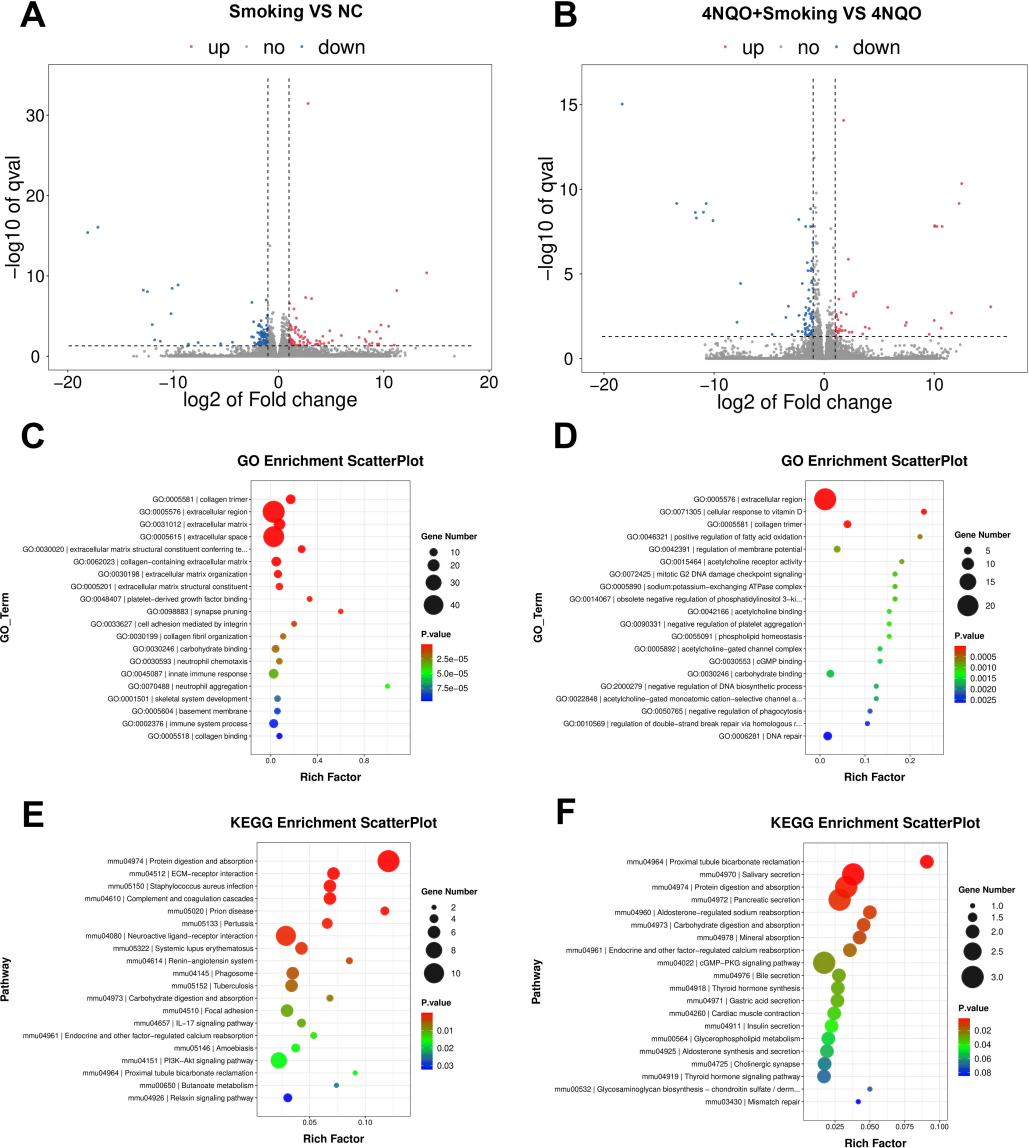
Sixteen-week RNA sequencing identified differentially expressed mRNAs between the groups. **(A, B)** Transcriptome volcano map. Differentially expressed genes between groups were identified using a default threshold of p < 0.05 and |log2FC| ≥ 1. The identified genes were then visualized on a map to represent their expression levels. Blue indicates downregulated genes, and red indicates upregulated genes. **(A)** Smoking vs. NC group and **(B)** 4NQO+Smoking vs. 4NQO group. **(C)** GO functional enrichment analysis in Smoking vs. NC group. **(D)** GO functional enrichment analysis in 4NQO+Smoking vs. 4NQO group. **(E)** KEGG enrichment analysis in Smoking vs. NC group. **(F)** KEGG enrichment analysis in 4NQO+Smoking vs. 4NQO group. 4NQO, 4-nitroquinoline-1-oxide; GO, Gene Ontology; KEGG, Kyoto Encyclopedia of Genes and Genomes.

In the 16-week groups, 87 upregulated genes and 106 downregulated genes were identified in the Smoking vs. NC group. In the 4NQO+Smoking vs. 4NQO group, 46 upregulated genes and 72 downregulated genes were identified (**Figures 3A, B**). GO enrichment analysis revealed that DEGs in the Smoking vs. NC group were primarily associated with collagen trimer, extracellular region, and extracellular matrix structural constituent conferring tensile strength (**Figure 3C**). In the 4NQO+Smoking vs. 4NQO group, DEGs were mainly enriched in the extracellular region, cellular response to vitamin D, and collagen trimer (**Figure 3D**). KEGG analysis showed that genes significantly enriched in the Smoking vs. NC group were mainly associated with Protein digestion and absorption, ECM–receptor interaction, Complement, and coagulation cascades (**Figure 3E**). In the 4NQO+Smoking vs. 4NQO group, genes were mainly associated with Proximal tubule bicarbonate reclamation, Salivary secretion, and Protein digestion and absorption signal pathway (**Figure 3F**). Similarly, the RNA-Seq analysis results of the 4NQO vs. NC group at 4 weeks and 16 weeks are shown in **Supplementary Figure 2**.

### 3.3 Bioinformatics analysis

**Figure 4:**
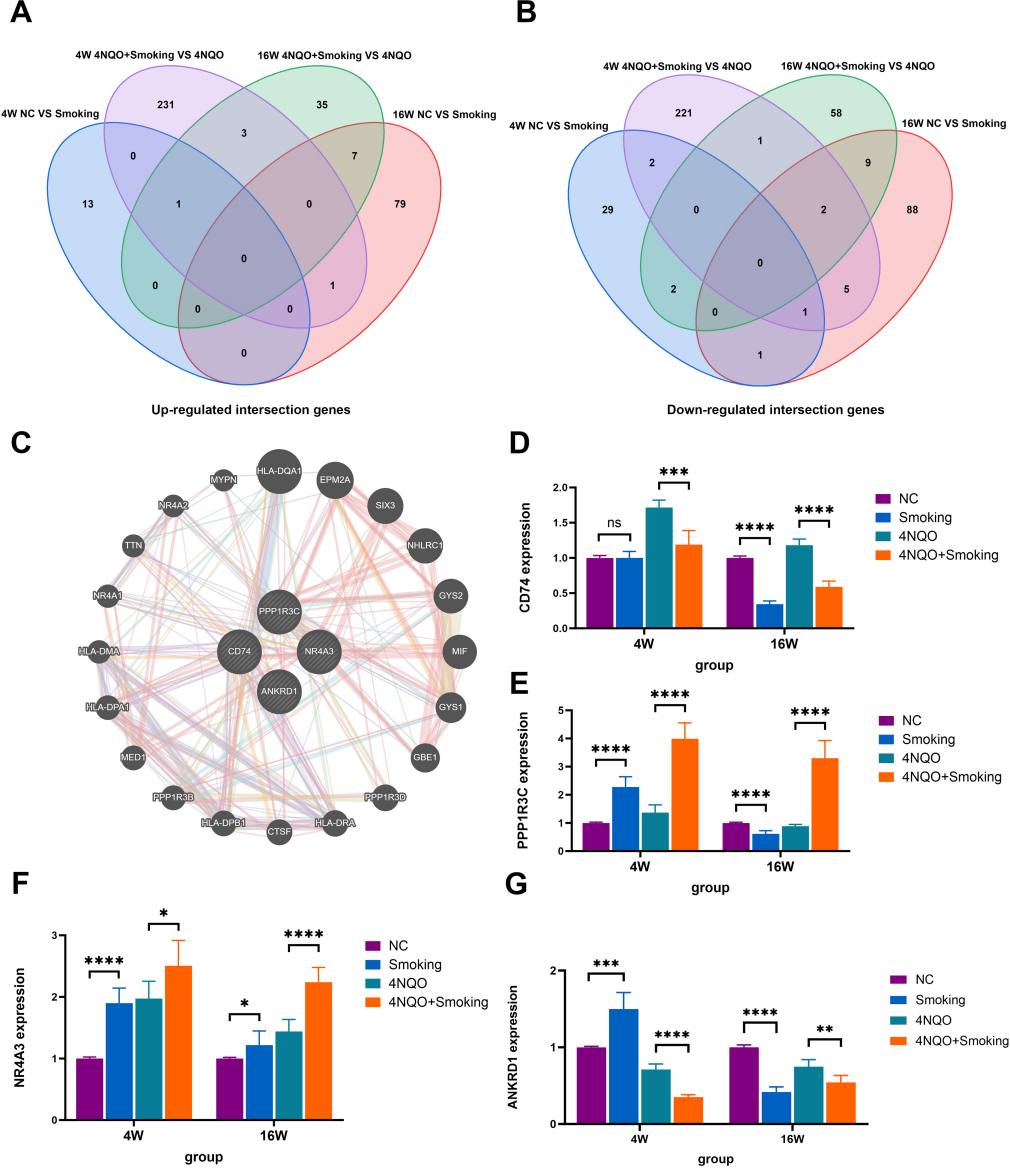
**(A)** Venn diagram comparing the number of upregulated genes among the four groups. **(B)** Venn diagram comparing the number of downregulated genes among the four groups. **(C)** The top 10 genes with the highest expression levels were entered into GeneMANIA database to obtain the PPI network diagram. **(D)** The mRNA expression of CD74. **(E)** The mRNA expression of PPP1R3C. **(F)** The mRNA expression of NR4A3. **(G)** The mRNA expression of ANRD1. *p < 0.05, **p < 0.01, ***p < 0.001 vs. control. PPI, protein–protein interaction.

Differentially expressed genes were identified by comparing the Smoking vs. NC group and the 4NQO+Smoking vs. 4NQO group at both 4 weeks and 16 weeks. Statistically significant upregulated and downregulated genes at two time points were combined and intersected, resulting in the identification of 12 upregulated and 23 downregulated crossover genes (**Figures 4A, B**). In total, 35 intersecting genes were ranked based on their expression levels. Four tumor-related genes (NR4A3, CD74, PPP1R3C, and ANKRD1) with high differential expression were selected, and the GeneMANIA database was used to generate a protein–protein interaction (PPI) network diagram (**Figure 4C**). The co-expression in the PPI network diagram is 77.64%, genetic interactions are 2.87%, co-localization is 8.01%, and the predicted value is 5.37%. Functional evaluation indicates that these genes are mainly involved in various metabolic pathways, positive regulation of white blood cell and lymphocyte proliferation, and regulation of related pathways by P53 class mediators. In addition, the same method was used to compare the 4NQO vs. NC group at 4 weeks and 16 weeks, and the results are shown in **Supplementary Figures 3A–C**.

### 3.4 RT-PCR

To validate the aforementioned four genes, RNA was extracted from tongue tissues and subjected to RT-PCR analysis. At 4 weeks, the expression levels of NR4A3, PPP1R3C, and ANKRD1 were significantly upregulated in the Smoking group compared to the NC group. Conversely, in comparison to the 4NQO group, the 4NQO+Smoking group exhibited significant upregulation of NR4A3 and PPP1R3C, while CD74 and ANKRD1 were downregulated. At 16 weeks, the expression of NR4A3 was significantly upregulated in the Smoking group compared to the NC group, whereas CD74, PPP1R3C, and ANKRD1 were significantly decreased. Similarly, compared to the 4NQO group, the 4NQO+Smoking group showed increased expression levels of NR4A3 and PPP1R3C, while CD74 and ANKRD1 levels were decreased (**Figures 4D–G**). The verification results of the 4NQO vs. NC group are shown in **Supplementary Figures 3D–O**.

### 3.5 Immunohistochemistry

**Figure 5:**
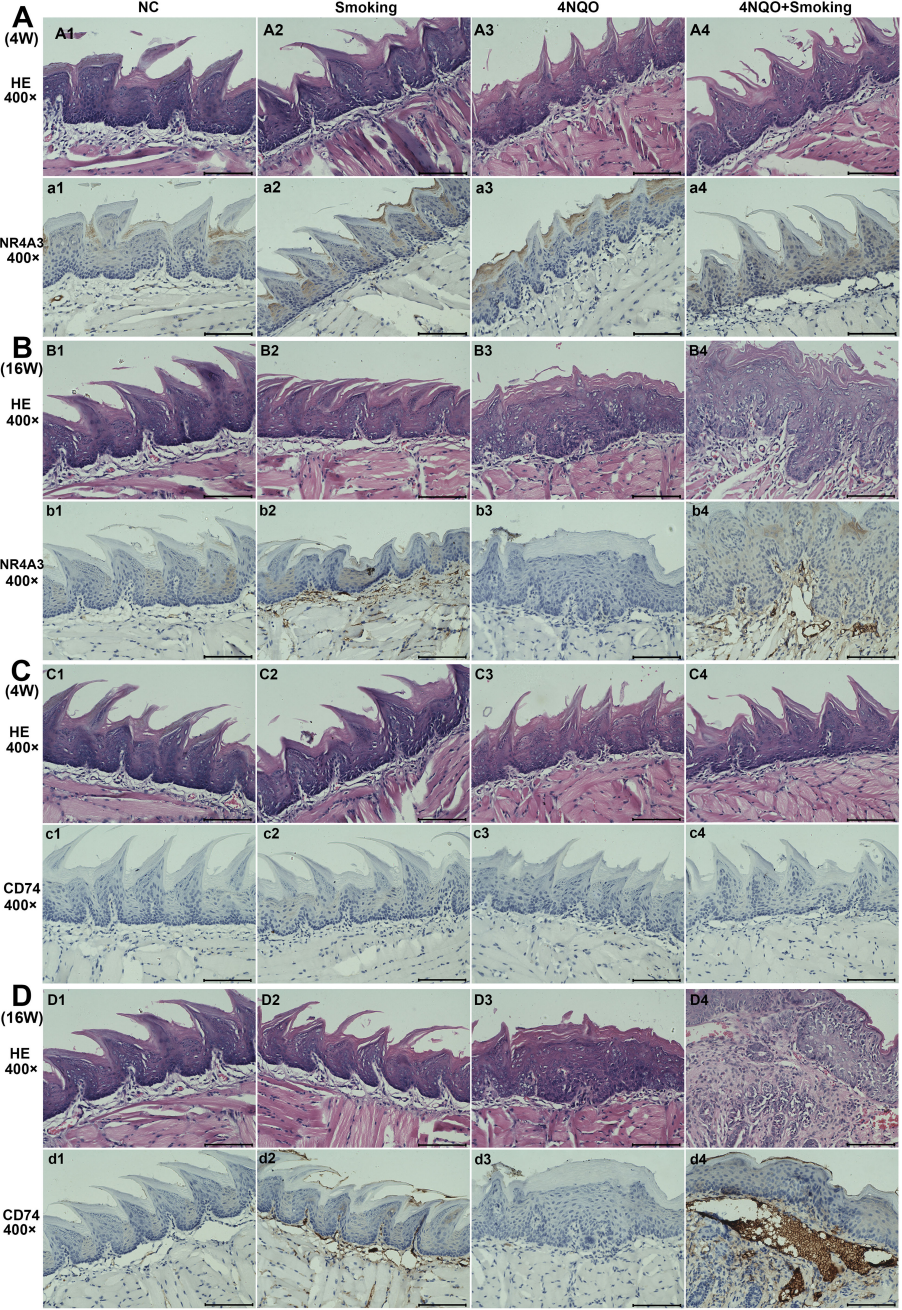
The results of H&E staining and IHC staining for NR4A3 and CD74 in four groups. **(A)** The expression of NR4A3 in 4-week tongue tissues. **(B)** The expression of NR4A3 in 16-week tongue tissues. **(C)** The expression of CD74 in 4-week tongue tissues. **(D)** The expression of CD74 in 16-week tongue tissues. Scale bar = 100 μm. IHC, immunohistochemistry.

We selected NR4A3 and CD74 for immunohistochemical validation. The results showed no positive expression of NR4A3 or CD74 at 4 weeks (**Figures 5A, C**). However, we found a small amount of NR4A3-positive expression in the cytoplasm of the submucosa in both the Smoking group and the 4NQO+Smoking group at 16 weeks. Additionally, we observed CD74-positive cells in the Smoking group and 4NQO+Smoking group, particularly in the 4NQO+Smoking group (**Figures 5B, D**).

### 3.6 Effects of smoking and/or 4NQO treatment on the oral microbiome during the development of OSCC in mice

#### 3.6.1 Data quality control

The average number of raw reads obtained from all samples was M ± N. After two-terminal splicing, quality control, and chimera filtering, the valid reads were m ± N. The average percentage of Valid% reads was M, with Q20% and Q30% values exceeding 97% and 91%, respectively.

#### 3.6.2 Alpha diversity analysis

**Figure 6:**
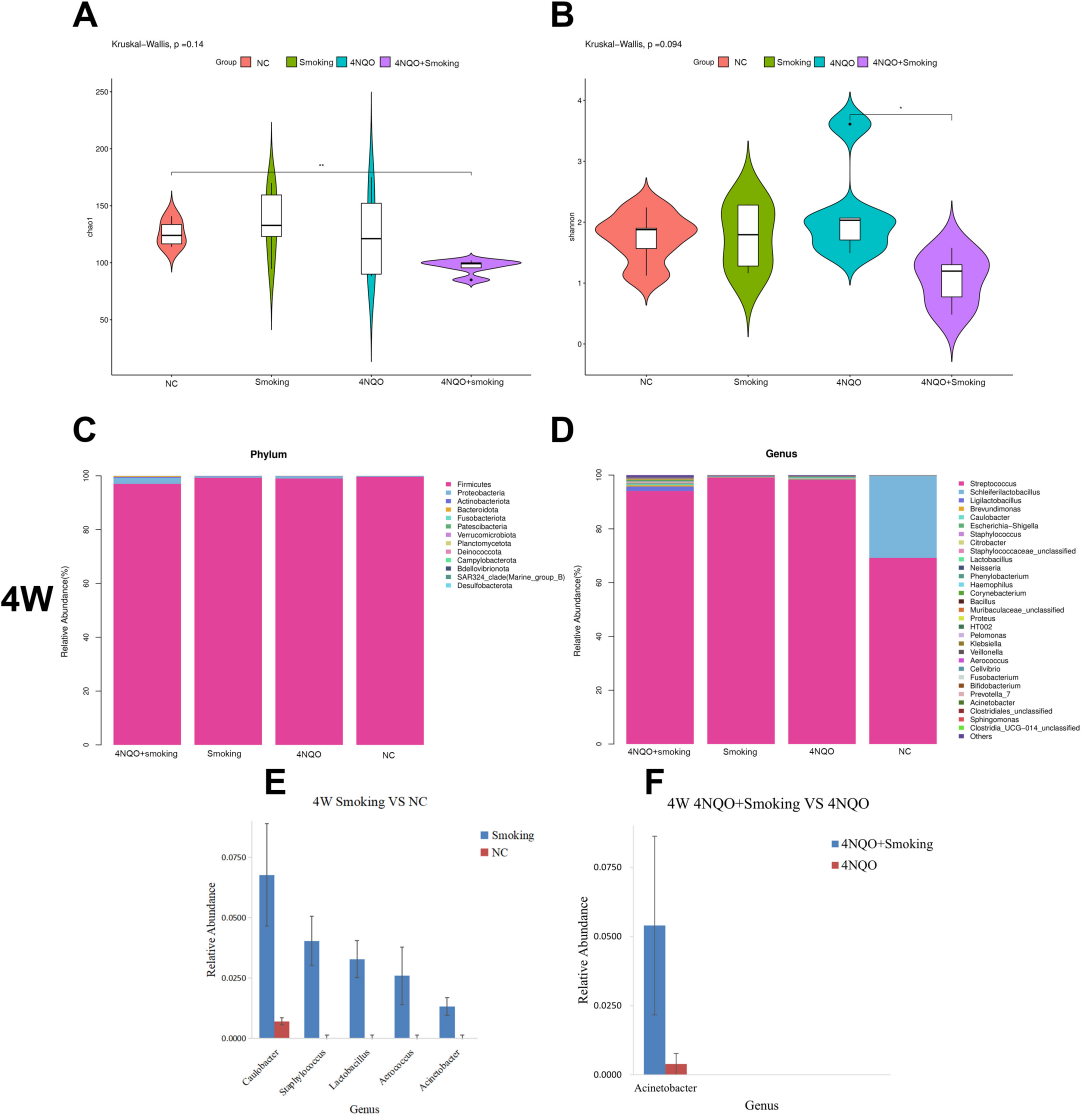
The composition of microbiome at different time points and groups. **(A)** Chao1 index at 16 weeks. **(B)** Shannon index at 16 weeks. **(C)** Relative abundance of phylum level in 4 weeks. **(D)** Relative abundance of genus level in 4 weeks. **(E, F)** Four-week statistically significant bacteria in Smoking vs. NC group at genus level **(E)** and 4NQO+Smoking vs. 4NQO group at genus level **(F)**. *p < 0.05, **p < 0.01 vs. control. 4NQO, 4-nitroquinoline-1-oxide.

The Chao1 index results indicated that the bacterial diversity and richness of bacteria in the 4NQO+Smoking group were significantly decreased compared to those in the NC group at 16 weeks. Additionally, the Shannon index in the 4NQO+Smoking group was lower than that in the 4NQO group (**Figures 6A, B**).

#### 3.6.3 Analysis of oral microbiome composition at 4 weeks

A stacked column chart was utilized to display the relative abundance of different levels in each group, facilitating visual comparison of sample abundances. The expression and trend of dominant bacteria across different groups at each taxonomic level were examined. The histogram and heatmap at the phylum and genus levels at 4 weeks are presented in **Figure 6**. At the phylum level, Firmicutes, Proteobacteria, and Actinobacteriota were the predominant bacteria in each group. Compared to that in the NC group, the relative abundance of Firmicutes decreased while Proteobacteria and Actinobacteriota increased in the 4NQO group (**Supplementary Figure 4A**). No significant differences in bacterial abundance were observed between the 4NQO+Smoking group and the 4NQO group (**Figure 6C**).

At the genus level, in comparison to the NC group, the Smoking group showed relative increases in *Caulobacter*, *Staphylococcus*, *Lactobacillus*, and *Acinetobacter*. Similarly, the 4NQO group exhibited relative increases in *Lactobacillus*, *Ligilactobacillus*, *Caulobacter*, and *Citrobacter* (**Supplementary Figure 4B**). The 4NQO+Smoking group demonstrated relative increases in *Ligilactobacillus*, *Caulobacter*, *Staphylococcus*, *Citrobacter*, *Lactobacillus*, and *Acinetobacter*, with a notably higher relative abundance of *Acinetobacter* compared to 4NQO group (**Figure 6D**). Statistical analysis using the Wilcoxon rank-sum test revealed significant changes at both the phylum and genus levels, with the results displayed in **Figures 6E, F**.

#### 3.6.4 Analysis of oral microbiome composition at 16 weeks

**Figure 7:**
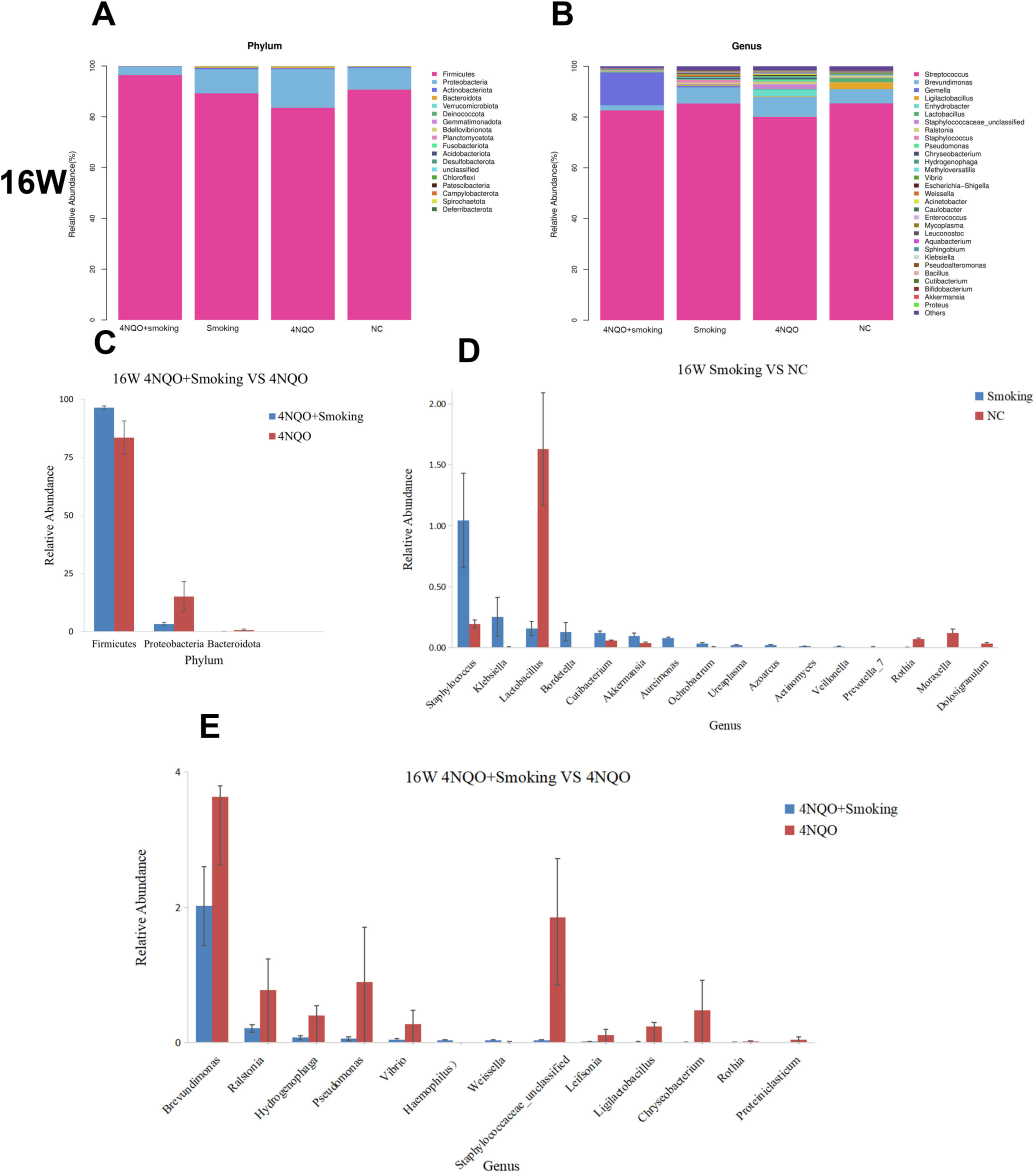
**(A)** Relative abundance of phylum level in 16 weeks. **(B)** Relative abundance of genus level in 16 weeks. **(C–E)** Sixteen-week statistically significant bacteria in 4NQO+Smoking vs. 4NQO group at phylum level **(C)**, Smoking vs. NC at genus level **(D)**, and 4NQO+Smoking vs. 4NQO group at genus level **(E)**. 4NQO, 4-nitroquinoline-1-oxide.

Histograms and heatmaps at the phylum and genus levels at 16 weeks are presented in **Figure 7** and **Supplementary Figure 4**. At the phylum level, Firmicutes, Proteobacteria, and Bacteroidota were the dominant bacteria in each group. There was no significant difference in bacterial abundance between the Smoking and NC groups (**Figure 7A**) or the 4NQO vs. NC group (**Supplementary Figure 4C**). In the 4NQO+Smoking group, the relative abundance of Firmicutes increased, and Proteobacteria and Bacteroidota decreased compared to that in the 4NQO group (**Figure 7A**).

At the genus level, compared to the NC group, *Staphylococcus*, *Klebsiella*, and Prevotella_7 relatively increased, whereas *Lactobacillus*, *Rothia*, and *Moraxella* decreased in the Smoking group (**Figure 7B**). The relative abundance of *Lactobacillus*, *Moraxella*, *Rothia*, and *Weissella* in the 4NQO group (**Supplementary Figure 4D**). In the 4NQO+Smoking group, the relative abundance of *Haemophilus* and *Weissella* was significantly higher, while the relative abundance of *Brevundimonas*, *Ralstonia*, *Pseudomonas*, and *Rothia* was lower than in the 4NQO group (**Figure 7B**). Statistical analysis using the Wilcoxon rank-sum test revealed significant changes at the phylum and genus levels, as shown in **Figures 7C–E** and **Supplementary Figures 4E–G**. In addition to the changes at the phylum and genus levels, bacterial changes at the order and family levels were also observed, suggesting bacterial alterations or disorders during the development of OSCC in mice.

#### 3.6.5 LEfSe analysis

**Figure 8:**
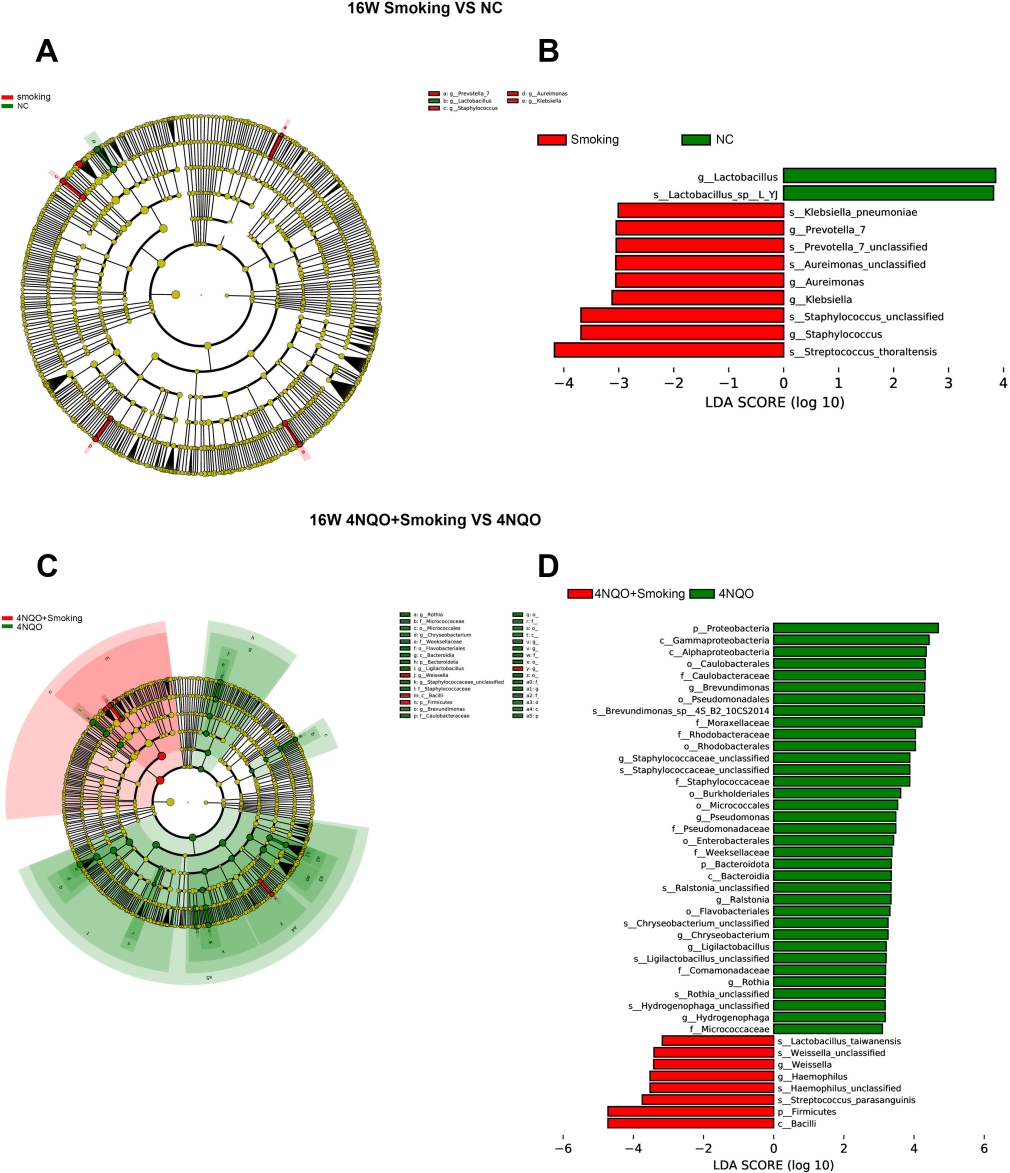
LEFSe analysis of the mice in each group at 16 weeks. **(A)** Species evolution branch diagram of the NC and Smoking groups. **(B)** Distribution of LDA values in the NC and Smoking groups. **(C)** Species evolution branch diagram of the 4NQO+Smoking and 4NQO groups. **(D)** Distribution of LDA values in the 4NQO+Smoking and 4NQO groups. LEFSe, linear discriminant analysis effect size; LDA, linear discriminant analysis.

To further explore these findings, the high-dimensional class comparison was performed *via* linear discriminant analysis effect size (LEfSe). Different abundant bacteria were found in the oral microbiomes between the 16-week groups. The analysis results are depicted in **Figures 8A, B**. In the Smoking vs. NC group, g_Lactobacillus was enriched in the NC group, and g_Prevotella_7, g_Aureimonas, g_Klebsiella, and g_Staphylococcus were enriched in the Smoking group. In the 4NQO+Smoking vs. 4NQO group, P_Proteobacteria, g_Brevundimonas, f_Moraxellaceae, g_Staphylococcaceae, g_Pseudomonas, p_Bacteroidota, and g_Ralstonia were enriched in the 4NQO group, and g_Weissella, g_Haemophilus, and p_Firmicutes were enriched in the 4NQO+Smoking group (**Figures 8A–D**).

### 3.7 Integrative analysis of oral microbiome and transcriptome

**Figure 9:**
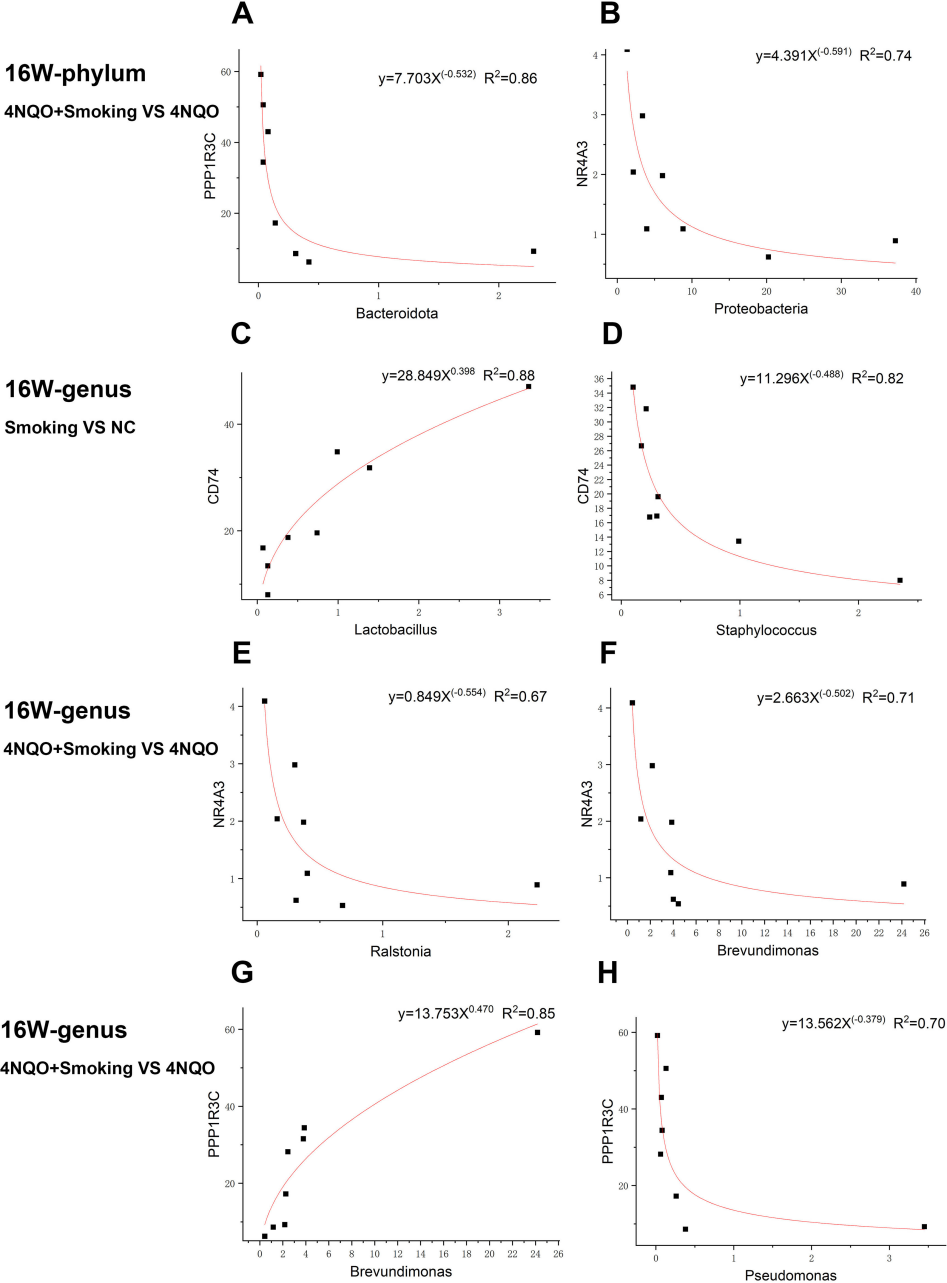
Representative gene–microbe correlations in smoking-associated OSCC with the strength of correlation (R^2^) indicated at the top. **(A, B)** Sixteen-week phylum-level fitting curve of PPP1R3C and Bacteroidota **(A)** and NR4A3 and Proteobacteria **(B)** in 4NQO+Smoking vs. 4NQO group. **(C, D)** Sixteen-week genus-level fitting curve of CD74 and *Lactobacillus* **(C)** and CD74 and *Staphylococcus* **(D)** in Smoking vs. NC group. **(E–H)** Sixteen-week genus-level fitting curve of NR4A3 and *Ralstonia* **(E)**, NR4A3 and *Brevundimonas* **(F)**, PPP1R3C and *Brevundimonas* **(G)**, and PPP1R3C and *Pseudomonas* **(H)** in 4NQO+Smoking vs. 4NQO group. OSCC, oral squamous cell carcinoma.

The allometric function was used to fit the DEGs and differential bacteria, revealing a non-linear relationship between them. At the phylum level, in the 16-week 4NQO+Smoking vs. 4NQO group, the expression of PPP1R3C was negatively correlated with the relative abundance of Bacteroidota. Similarly, NR4A3 expression was negatively correlated with the relative abundance of Proteobacteria (**Figures 9A, B**).

At the genus level, in the 16-week Smoking vs. NC group, the expression of CD74 was positively correlated with the relative abundance of *Lactobacillus* but negatively correlated with the relative abundance of *Staphylococcus* (**Figures 9C, D**). In the 16-week 4NQO+Smoking vs. 4NQO group, the expression of NR4A3 was negatively correlated with the relative abundance of *Brevundimonas* and *Ralstonia*, while PPP1R3C expression was positively correlated with the relative abundance of *Brevundimonas* and negatively correlated with that of *Pseudomonas* (**Figures 9E–H**).

## 4 Discussion

As the second most complex microbial ecosystem after the intestinal flora (19), the interaction between oral bacteria and their host affects various physiological processes and can contribute to cancer occurrence, progression, and invasion (20). The oral microbiome is influenced by OSCC risk factors such as smoking, alcohol abuse, and human papillomavirus (HPV) infection (21), among which smoking has been a key focus of oral mucosa research for a long time (22, 23). As the first tissue exposed to cigarette smoke, the oral mucosa is affected by toxic substances in the smoke, which promote the development of various oral diseases by impacting immunity and inflammation. Yanan Zhu et al. found in 4NQO and smoking mouse models that smoking can promote the formation of oral leukoplakia in mice by regulating glutamine metabolism and macrophage M2 polarization (7), providing a reference for studying the effect of smoking on OSCC in mice.

4NQO is an aromatic amine heterocyclic compound that metabolizes into an electrophilic form in the body and irreversibly reacts with nucleophilic sites on DNA, thereby altering gene expression.

The continuous morphological changes in the oral epithelium of mice treated with 4NQO are similar to those observed during the progression of human OSCC (24). However, 4NQO is not sterile and may affect the oral microbiome. Few studies have reported the effects of 4NQO itself on the oral microbiome, making it difficult to quantify which changes in the 4NQO+Smoking group are caused by 4NQO. Therefore, we established a separate 4NQO group as a control in this study to isolate the changes caused by 4NQO in the 4NQO+Smoking group, thereby reducing the confounding effects of 4NQO. Furthermore, we also compared the results of the 4NQO group and NC group to observe the effect of 4NQO itself.

In recent years, the importance of host–microbiome interactions in the pathogenesis of OSCC has become increasingly evident (14). In this study, although no histological changes were observed at 4 weeks, we found alterations in cellular transcriptional levels and the oral microbiome. At 16 weeks, abnormal epithelial hyperplasia appeared, accompanied by more pronounced changes in gene expression and bacterial composition. Although the relationship between host gene transcription and microbial changes in OSCC has rarely been reported, it has been studied in other diseases such as cystic fibrosis. This suggested that communication between the host and microbiome may depend on specific genes, with the interaction being bi-directional—the microbiome influencing host gene expression—and host genes, in turn, forming the habitat for the microbiome (25). To understand the role of smoking–host–microbiome interactions in OSCC, we jointly profiled tongue gene expression and microbiome composition data in mice. We found a correlation between DEGs and differential bacteria, including NR4A3 with Proteobacteria and CD74 with *Staphylococcus*.

Proteobacteria is one of the most abundant and heterogeneous bacterial groups in the oral cavity, with its abundance only surpassed by that of Firmicutes and Bacteroides (26). Therefore, it plays a crucial role in maintaining the stability of the oral microenvironment. Current reports indicate that the relative abundance of Proteobacteria decreased as OSCC progressed (27–29). However, there is limited information about the specific role of Proteobacteria in OSCC. In this study, we found that the relative abundance of Proteobacteria in the 4NQO+Smoking group was lower compared to that in the 4NQO group at 16 weeks. The expression of NR4A3 was negatively correlated with Proteobacteria. NR4A3 is a nuclear receptor and transcription factor involved in various cellular, metabolic, and tumor inhibition processes (30). Our RNA-Seq, qRT-PCR, and IHC results showed that NR4A3 was upregulated in both the Smoking vs. NC group and the 4NQO+Smoking vs. 4NQO group. As reported, NR4A3 regulates the transcription of overlapping target genes and may serve as a balance regulator of proliferation, apoptosis, and differentiation, garnering significant attention in cancer research. In acinar cell carcinoma, the spatial proximity of the secretory calcium-binding phosphorylation protein gene cluster to the transcription start site of NR4A3 on chromosome 9 initiates the upregulation of CCND1 and ENO3 genes, promoting acinar cell carcinoma through cell proliferation and cell cycle (31). Moreover, NR4A3 has been identified as one of the diagnostic biomarkers for acinar cell carcinoma due to its high sensitivity and specificity. However, whether its high expression and the low expression of Proteobacteria can also serve as early detection markers of OSCC remains a topic for further study.

*Staphylococcus* is one of the most common oral and perioral bacteria (32). It can cause a range of diseases, from wound infections to fatal sepsis or multi-organ failure (33). Studies have shown that *Staphylococcus* enterotoxin C1 can inhibit the growth of bladder cancer (34), while its lipoteichoic acid can promote the proliferation of lung cancer cells (35). This indicates a strong relationship between *Staphylococcus* and cancer. In oral cancer, *Staphylococcus* has been found to upregulate the fnbpB gene, activating the Cyclooxygenase (COX-2)/prostaglandin E2 pathway in oral epithelial cells, thereby promoting the development of OSCC. However, its specific mechanism remains unclear (36). In this study, the expression of CD74 was found to be negatively correlated with *Staphylococcus*. CD74 is a receptor for the tumor cytokine macrophage migration inhibitory factor (MIF) (37). The interaction between MIF and CD74 can trigger various signaling pathways related to tumor cell survival and proliferation, such as PI3K/AKT and NF-κB pathways, playing a downstream role in cell survival and proliferation (38). Through IHC, we observed a significant positive expression of CD74 in the Smoking group and 4NQO+Smoking group, indicating that smoking may increase CD74 expression. In addition to its expression in immune cells, CD74 is highly expressed in various tumor cells. In head and neck squamous cell carcinoma, CD74 expression is significantly increased during tumor progression (39). However, our RNA-Seq and RT-PCR results showed a downward trend in the Smoking vs. NC group and 4NQO+Smoking vs. 4NQO group. We speculate that this opposite result may be due to post-transcriptional modifications in mRNA, but the specific mechanism requires further exploration.

In the early development of smoking-related OSCC, differential genes are primarily associated with inflammation, immunity, metabolism, cell proliferation, and other processes. These genes are predominantly enriched in pathways related to protein digestion and absorption, retinol metabolism, ECM–receptor interaction, circadian rhythm, cell adhesion molecules, and the PI3K/AKT signaling pathway. Among these, ECM–receptor interaction (40) and the PI3K/AKT (41, 42) signaling pathway have been implicated in OSCC development.

The changes in the oral microbiome may contribute to the development of oral cancer. Conversely, bacterial alterations could also reflect responses to environmental changes in the oral cavity due to malignancy (43). However, we found notable differences in the oral microbiome between mice and humans. At the genus level, the predominant bacteria in the 4NQO and 4NQO+Smoking groups of mice included *Streptococcus*, *Ligilactobacillus*, *Brevundimonas*, *Gemella*, *Lactobacillus*, and *Staphylococcus*. However, previous studies have reported that *Fusobacterium*, *Streptococcus*, *Prevotella*, *Rotella*, and *Neisseria* are more prevalent in human oral OSCC tissues (29, 44, 45). This indicates that although we observed dynamic changes in the microbiome during tumorigenesis, the oral microbiomes of mice and humans are significantly different (46–48). One reason for this disparity may be that mice are scavengers, leading to the presence of numerous intestinal organisms in their oral microbiome (46). Therefore, further refinement of the model may be necessary in future studies.

In this study, RNA-Seq and 16S rDNA sequencing technologies, combined with RT-PCR and IHC, were used to explore gene transcription and changes in the oral microbiome during the development of smoking-related OSCC in mice. A correlation between genes and bacteria was identified, providing a new avenue for further research. However, several limitations remain: first, there may be differences in oral microbiome and gene expression patterns between mice and humans, making the translation of research from mice to humans challenging (48, 49). In future studies, we plan to first stimulate the human oral microecological environment in the mice oral cavity, then construct the 4NQO and/or smoking model, and verify the differential genes and bacteria using human tissue samples. Second, the sample size and the number of observed time points in this experiment may be limited, potentially generating random errors or overlooking some crucial changes. We will improve the experiment protocol, expand the sample size based on power analysis, and design a series of time points to obtain more accurate results. Finally, although gene–microbial correlations have been identified, there is a lack of mechanism studies between them, and the RNA-Seq verification methods are also limited. We will further elucidate the mechanisms through functional experiments such as cell experiments and use additional methods to verify the sequencing results in subsequent studies.

In summary, this study was a preliminary exploration of the host–microbial regulatory relationship in the development of smoking-related OSCC in mice, laying a foundation for subsequent research. The aforementioned limitations will be addressed in future studies.

## 5 Conclusion

In conclusion, we have conducted an analysis of the oral microbiome and host gene expressions in the development of smoking-related OSCC. Our findings indicate that differential genes primarily play roles in metabolism and immune response and cause inflammation. Furthermore, we observed variations in the microbiome that may contribute to dysbiosis and an increase in pathogenic strains.

Importantly, we identified interactions between host genes and the oral microbiome. Our study highlights the significance of host–microbiome interactions and their correlation in the early development of OSCC. These results may provide researchers and clinicians with potential biomarkers for the early diagnosis and prevention of OSCC.

## Data Availability

The data presented in the study are deposited in the NCBI repository, accession number PRJNA1145554, PRJNA1145571, PRJNA1145662, PRJNA1145877.
