# Supplementary material for: Integration of host gene regulation and oral microbiome reveals the influences of smoking during the development of oral squamous cell carcinoma
Source: Front Oncol. 2024 Oct 15;14:1409623. doi: 10.3389/fonc.2024.1409623 (PMC11518844; doi:10.3389/fonc.2024.1409623)
Supplement: Supplementary file 1 [file DataSheet1.docx]

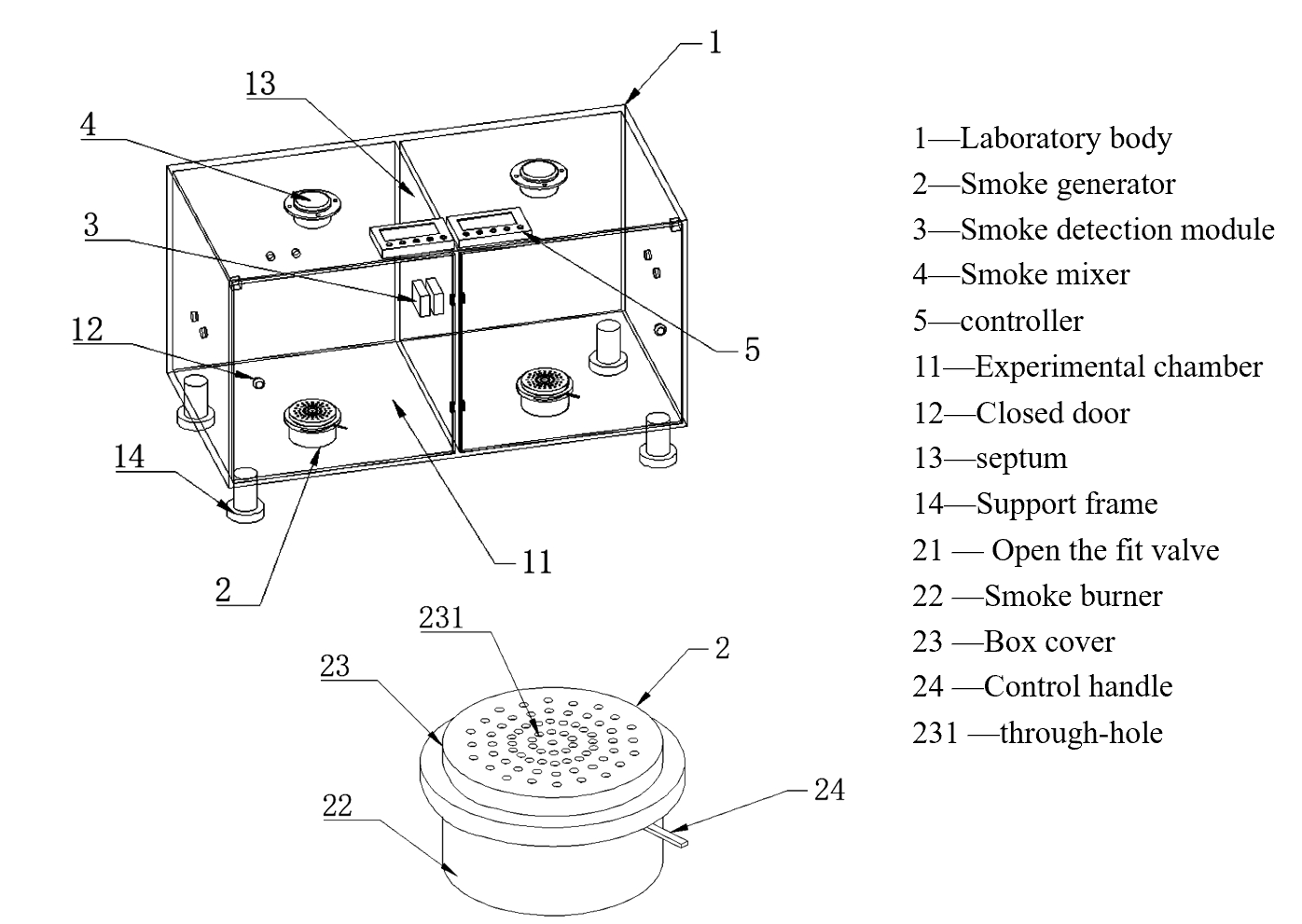


Fig.S1 Mice smoking special device

(Chinese utility model patent number: ZL202223479539.9)


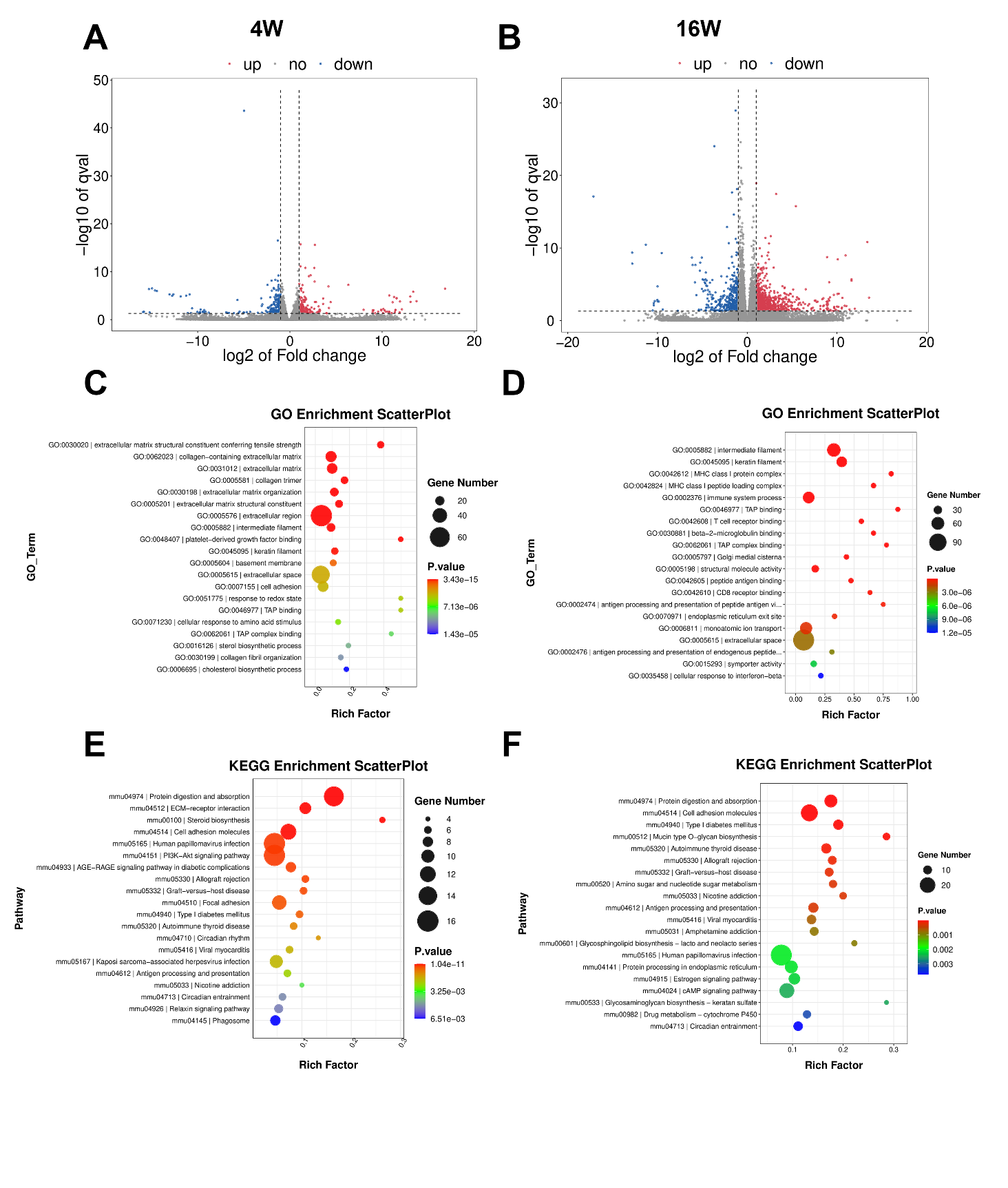


Fig.S2 Transcriptome sequencing results. (A, B) Transcriptome volcano map. Differentially expressed genes between groups were identified using a default threshold of P<0.05 and |log2FC|≥1. The identified genes were then visualized on a map to represent their expression levels. Blue indicates down-regulated genes, and red indicates up-regulated genes. (A) 4W 4NQO VS NC group; (B) 16W 4NQO VS NC group. (C) GO functional enrichment analysis in 4W 4NQO VS NC group; (D)GO functional enrichment analysis in 16W 4NQO VS NC group. (E) KEGG enrichment analysis in 4W 4NQO VS NC group; (F) KEGG enrichment analysis in 16W 4NQO VS NC group.


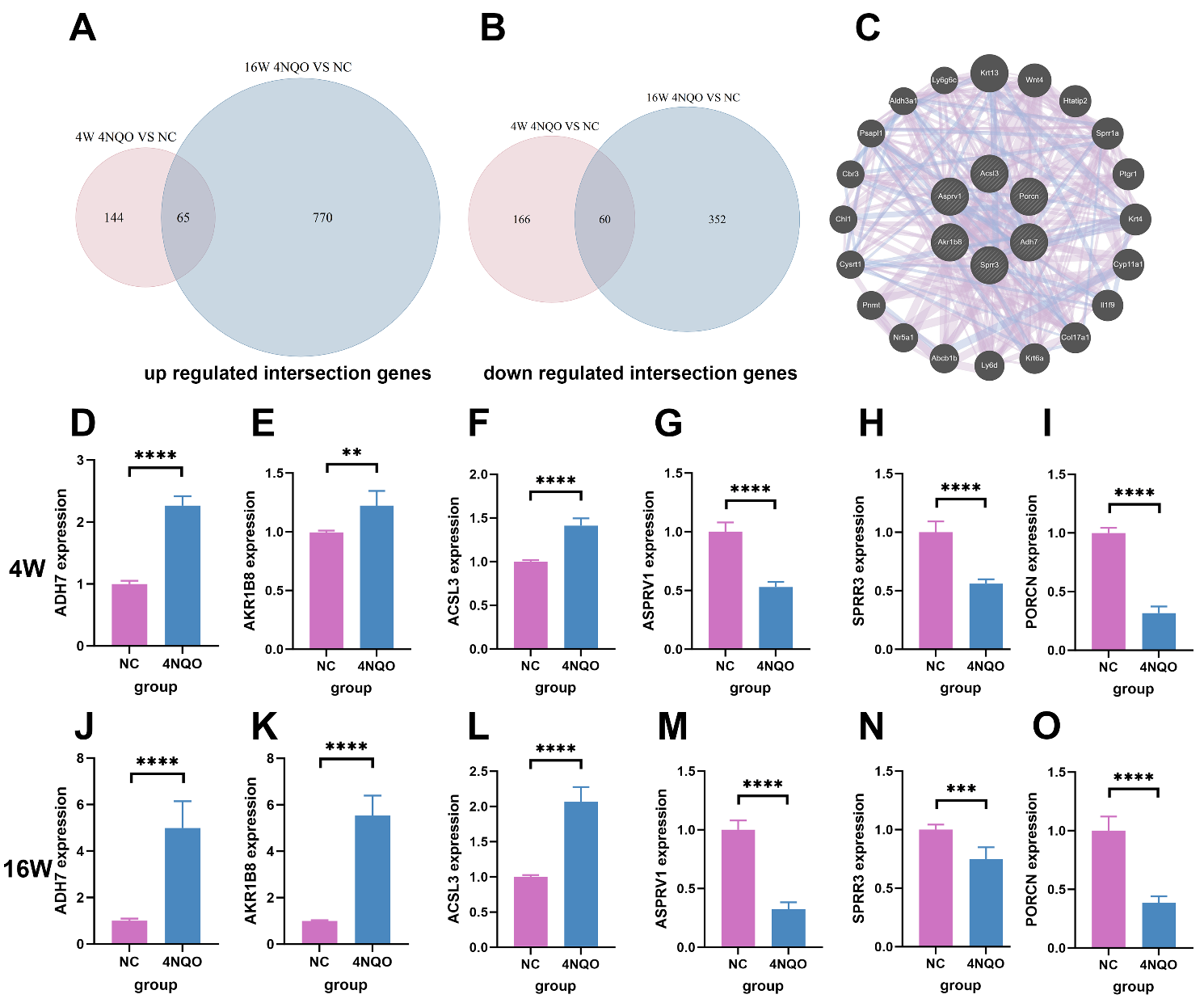


Fig. S3 (A)Venn diagram comparing the number of upregulated genes among the groups. (B) Venn diagram

Comparing the number of downregulated genes among the groups. (C) The top genes with the highest expression levels and tumor-related were entered into GeneMANIA database to obtain the PPI network diagram. (D) The mRNA expression of ADH7 at 4-week. (E) The mRNA expression of AKR1B8 at 4-week. (F) The mRNA expression of ACSL3 at 4-week. (G) The mRNA expression of ASPRV1 at 4-week. (H) The mRNA expression of SPRR3 at 4-week. (I) The mRNA expression of PORCN at 4-week. (J) The mRNA expression of ADH7 at 16-week. (K) The mRNA expression of AKR1B8 at 16-week. (L) The mRNA expression of ACSL3 at 16-week. (M) The mRNA expression of ASPRV1 at 16-week. (N) The mRNA expression of SPRR3 at 16-week. (O) The mRNA expression of PORCN at 16-week. *P＜0.05，**P＜0.01， ***P＜0.001 vs. control.


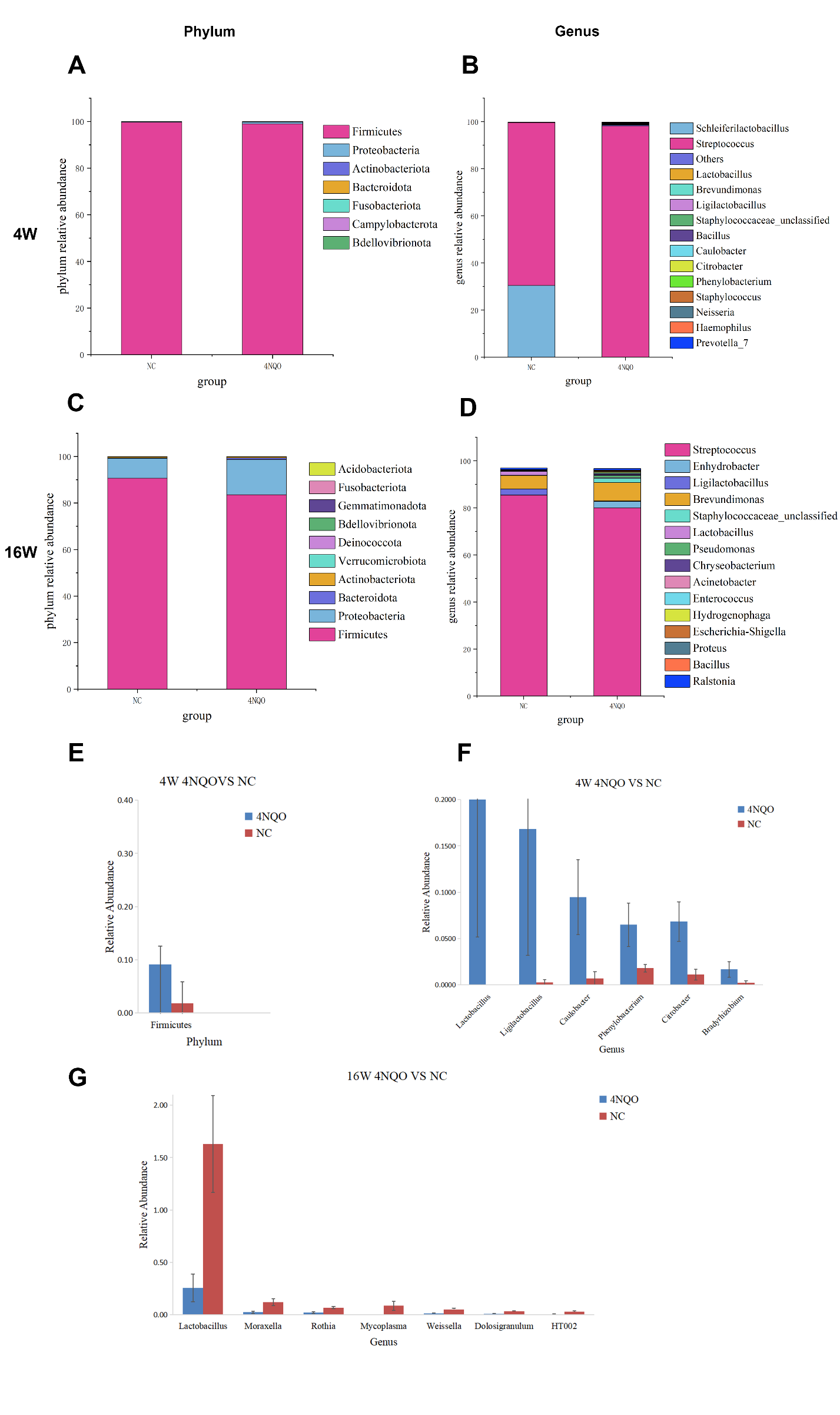


Fig.S4 (A) Relative abundance of phylum-level in 4-week. (B) Relative abundance of genus-level in 4-week. (C) Relative abundance of phylum-level in 16-week. (D) Relative abundance of genus-level in 16-week. (E) statistically significant bacteria at 4W-phylum-level. (F) statistically significant bacteria at 4W-genus-level. (G) statistically significant bacteria at 16W-genus-level.
